# Editorial: Psychometrics in psychiatry 2022: adolescent and young adult psychiatry

**DOI:** 10.3389/fpsyt.2024.1381171

**Published:** 2024-03-04

**Authors:** Tjhin Wiguna, Mohsen Khosravi

**Affiliations:** ^1^ Child and Adolescent Psychiatry Division, Department of Psychiatry, Faculty of Medicine Universitas Indonesia, Dr. Cipto Mangunkusumo General Hospital, Jakarta, Indonesia; ^2^ Department of Psychiatry, School of Medicine, Zahedan University of Medical Sciences, Zahedan, Iran

**Keywords:** measurement tool, psychiatry, adolescent, young adult, validity, reliability, validation

Psychometric properties in psychiatric measurement tools are designed to quantify human characteristics and enhance our understanding of complex behaviors, particularly in adolescent and young adult population (1). Adolescence is a period of turmoil, characterized not only by biological changes but also by evolving psychosocial competencies. The prefrontal cortex, which continues to develop during this time, influences rational decision-making. Concurrently, adolescents struggle to adapt to environmental challenges, notably in forming their identities. Moreover, during this critical period, adolescents intensely seek understanding and acceptance from their peers and environment. Consequently, they may experience feelings of inferiority, inadequate self-esteem, and a lack of self-confidence (2, 3). These conditions can affect adolescents’ mental health literacy, communication styles, and interpersonal relationships, including those with mental healthcare professionals. Therefore, psychiatric measurement tools for the adolescent and young adult population are invaluable for clinical purposes—such as early detection, identification, quantification of the severity of mental health problems/disorders and setting therapeutic goals—as well as for research.

It is estimated that adolescents comprise approximately 16% of the global population, with 89% residing in low- and middle-income countries (LMICs) (4). Mental health problems and disorders are estimated to affect 10–20% of adolescents and young adults globally, accounting for approximately 13% of the global burden of disease in this age group (5). Behavioural and emotional problems, such as depression, anxiety, and conduct issues, are the primary mental health concerns among adolescents and young adults, leading to increased disability within this population. Furthermore, undetected and inadequately managed mental health issues may persist into adulthood, diminishing the opportunities for fulfilling adult lives. Therefore, early detection and optimal treatment are crucial. In these cases, using measurement tools with excellent psychometric properties offers significant advantages.

Psychometric properties pertain to the validity and reliability of measurement tools. Before asserting that tools such as questionnaires or subtests have excellent psychometric properties, they should be evaluated extensively (1). Validity refers to the confirmation and certification of the accuracy of results or decisions generated from measurement tools. High validity means the tool produces results that correspond to the actual characteristics or variations of individuals. There are several types of validity: 1. Construct validity assesses how accurately the tool measures the concept it is intended to measure. 2. Content validity indicates the extent to which the tool represents what it aims to measure. 3. Face validity pertains to the appropriateness of the tool’s content for its intended purpose. 4. Criterion validity relates to the tool’s ability to quantify concrete outcomes as designed. 5. Concurrent validity demonstrates how closely a new tool’s results compare to those from well-established (standardized or “gold standard”) (Jusienė et al.) tools, with verification occurring simultaneously with the target measurement (6). Predictive validity includes sensitivity and specificity. Sensitivity is the measurement tool’s ability to detect adolescent or young adult with a particular condition (e.g., young adults with psychosocial distress) (Fong et al.), while specificity refers to the tool’s ability to accurately identify individuals without specific conditions (e.g., adolescents without tic disorder) (Che et al.) (7). Cross-cultural validity pertains when a measurement tools are culturally translated into different forms and languages, etc.

Meanwhile, reliability refers to how consistently a measurement tool assesses what it is intended to measure. It can be explained that when the same tools are applied to measure specific conditions in the same subject under the same circumstances, the tools should yield identical results. If they do not, the measurement tools may be unreliable or biased. There are four types of measurement tool reliability: (1) test-retest reliability, which shows the consistency of the same measurement tools over time; (2) interrater reliability, which refers to the consistency of the measurement tools even when applied by different individuals; (3) internal consistency, which measures the stability of the individual items within the tools and; (4) parallel forms reliability, which ensures that different versions of measurement tools are designed consistently and equivalently.

Reflecting on the aforementioned explanation, it is clear that psychometrically sound measurement tools in adolescent and young adult are invaluable for clinical practice, both in hospital and community settings. Currently, numerous psychiatric measurement tools have been developed and adapted into various languages and formats, such as Internet-based questionnaires or digital forms. These adaptations aim to improve screening, prediction, evaluation, and understanding of behavioural and emotional problems among adolescents and young adults. Notable examples include the validated Italian version of the Sociocultural Attitudes Towards Appearance Questionnaire-Social Media (SATAQ-SM), the factorial and convergent validity and measurement invariance of the Patient Health Questionnaire-4 (PHQ-4) in young adults in Hong Kong (Fong et al.), and the Chinese version of the Individualized Premonitory Urge for Tics Scale (C-IPUTS) (Jusienė et al.) which demonstrated good validity and reliability. Additionally, the Strengths and Difficulties Questionnaire-Self Report (SDQ-SR) (7) was good enough psychometric properties that are particularly suitable for the adolescent population, regardless of their educational background.

In conclusion, designing studies with robust psychometric properties is essential for psychiatric measurement tools aimed at adolescents and young adults. To enhance these studies, several key steps should be implemented. The first step involves creating an evidence-based culture to establish the validity of psychiatric measurement tools. This includes developing appropriate design rules, trial blueprinting, and standard setting before measurement. The second step requires reporting trial results considering reliability, assessors, demographic characteristics, and longitudinal analyses across multiple settings. The third step involves refining and selecting items or subtests through qualitative and quantitative analyses to improve the tools’ psychometric properties. The fourth step is conducting exploratory and confirmatory factor analyses to identify the underlying constructs of the measurement tools. Finally, continual re-evaluation of the psychometric characteristics ensures that the measurement tools remain relevant and applicable. By implementing these steps, studies of psychiatric measurement tools can yield more accurate and reliable measurements (Che et al.).

## Author contributions

TW: Writing – original draft, Writing – review & editing. MK: Writing – review & editing.
